# Bis{1-[(1*H*-benzimidazol-2-yl)meth­yl]-1*H*-imidazole-κ*N* ^3^}­bis­(3,5-dicarb­oxy­benzoato-κ*O* ^1^)zinc octa­hydrate

**DOI:** 10.1107/S160053681200147X

**Published:** 2012-01-18

**Authors:** Lei Zhao, Bingtao Liu, Ting Li, Xiangru Meng

**Affiliations:** a School of Chemical and Engineering, Zhengzhou University, Zhengzhou 450001, People’s Republic of China; b School of Environmental and Municipal Engineering, North China Institute of Water Conservancy and Hydroelectric Power, Zhengzhou 450011, People’s Republic of China; c Department of Chemistry, Zhengzhou University, Zhengzhou 450001, People’s Republic of China

## Abstract

In the title complex, [Zn(C_9_H_5_O_6_)_2_(C_11_H_10_N_4_)_2_]·8H_2_O, the Zn^II^ ion exhibits site symmetry 2. It shows a distorted tetra­hedral coordination defined by two N atoms from two symmetry-related 1-[(1*H*-benzimidazol-2-yl)meth­yl]-1*H*-imid­azole ligands and by two O atoms from two symmetry-related monodeprotonated 3,5-dicarb­oxy­benzoate anions. In the crystal, complex mol­ecules and solvent water mol­ecules are linked through inter­molecular O—H⋯O, O—H⋯N, and N—H⋯O hydrogen bonds into a three-dimensional network.

## Related literature

For background information on Zn^II^ complexes constructed from both aromatic carboxyl­ates and *N*-heterocyclic ligands, see: Lin *et al.* (2008 ▶); Tian *et al.* (2010 ▶).
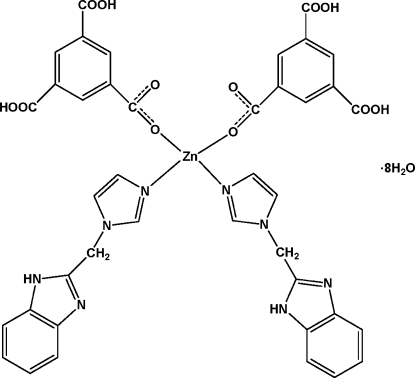


## Experimental

### 

#### Crystal data

[Zn(C_9_H_5_O_6_)_2_(C_11_H_10_N_4_)_2_]·8H_2_O
*M* *_r_* = 1024.22
Monoclinic, 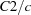

*a* = 20.870 (4) Å
*b* = 15.008 (3) Å
*c* = 15.472 (3) Å
β = 109.51 (3)°
*V* = 4567.9 (16) Å^3^
*Z* = 4
Mo *K*α radiation
μ = 0.63 mm^−1^
*T* = 293 K
0.18 × 0.15 × 0.14 mm

#### Data collection

Rigaku Saturn diffractometer
Absorption correction: multi-scan (*CrystalClear*; Rigaku/MSC, 2004 ▶) *T*_min_ = 0.896, *T*_max_ = 0.917
15325 measured reflections
4127 independent reflections
3898 reflections with *I* > 2σ(*I*)
*R*_int_ = 0.031

#### Refinement

*R*[*F* ^2^ > 2σ(*F* ^2^)] = 0.054
*wR*(*F* ^2^) = 0.158
*S* = 1.07
4127 reflections
314 parameters
H-atom parameters constrained
Δρ_max_ = 1.08 e Å^−3^
Δρ_min_ = −0.45 e Å^−3^

### 

Data collection: *CrystalClear* (Rigaku/MSC, 2004 ▶); cell refinement: *CrystalClear*; data reduction: *CrystalClear*; program(s) used to solve structure: *SHELXS97* (Sheldrick, 2008 ▶); program(s) used to refine structure: *SHELXL97* (Sheldrick, 2008 ▶); molecular graphics: *SHELXTL* (Sheldrick, 2008 ▶); software used to prepare material for publication: *publCIF* (Westrip, 2010 ▶).

## Supplementary Material

Crystal structure: contains datablock(s) global, I. DOI: 10.1107/S160053681200147X/ff2052sup1.cif

Structure factors: contains datablock(s) I. DOI: 10.1107/S160053681200147X/ff2052Isup2.hkl

Additional supplementary materials:  crystallographic information; 3D view; checkCIF report

## Figures and Tables

**Table 1:** Hydrogen-bond geometry (Å, °)

*D*—H⋯*A*	*D*—H	H⋯*A*	*D*⋯*A*	*D*—H⋯*A*
O8—H8*A*⋯O9	0.85	2.22	2.936 (8)	142
O9—H9*A*⋯O4	0.85	2.15	2.634 (7)	115
O9—H9*B*⋯O10	0.85	2.24	2.783 (12)	122
O10—H10*A*⋯O9	0.85	1.93	2.783 (12)	179
N4—H4⋯O7^i^	0.86	1.97	2.808 (4)	163
O3—H3⋯O10^ii^	0.82	2.56	3.341 (10)	159
O5—H5⋯N3^iii^	0.82	1.77	2.577 (3)	165
O7—H7*A*⋯O2^iv^	0.85	1.98	2.784 (4)	156
O7—H7*B*⋯O8^v^	0.85	1.95	2.796 (5)	179
O8—H8*B*⋯O6^vi^	0.85	2.02	2.799 (6)	152
O10—H10*B*⋯O6^vii^	0.85	2.03	2.728 (9)	139

Symmetry codes: (i) 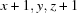; (ii) 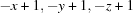; (iii) 
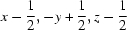
; (iv) 
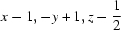
; (v) 
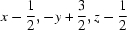
; (vi) 
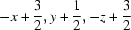
; (vii) 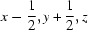.

**Fig. 1.**
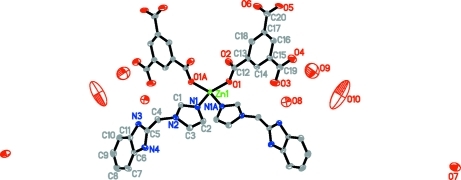
View of the title complex showing labeling and 30% probability displacement ellipsolids. H atoms are omitted for clarity. [Symmetry code A: -x + 2, +y, -z + 1.5.]

**Fig. 2.**
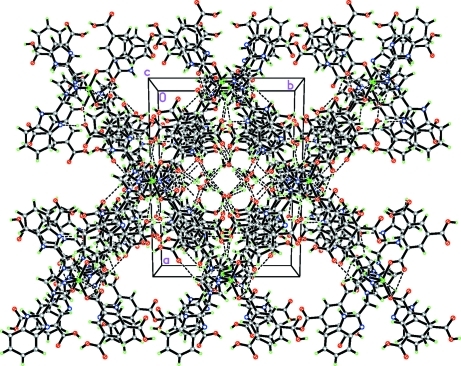
View of the crystal packing of the title complex, showing the three-dimensional structure stabilized by hydrogen bonds.

## supplementary crystallographic information

### Comment

It is well known that the Zn^II^ ion is able to coordinate simultaneously to both oxygen-containing and nitrogen-containing ligands and the final products can exhibit attractive structures and useful functional properties. A great number of Zn^II^ complexes containing both aromatic carboxylates and N-heterocyclic ligands have been reported (Lin *et al.*, 2008; Tian *et al.*, 2010). In order to further explore such compounds with new structures, we selected 1-((1*H*-benzimidazol-1-yl)methyl)-1*H*-imidazole and 1,3,5-benzenetricarboxylic acid as ligands to self-assemble with Zn(NO_3_)_2_ and obtained the title complex, {[Zn(C_9_H_5_O_6_)_2_(C_11_H_10_N_4_)_2_] (H_2_O)_8_}, the crystal structure of which is reported herein. As shown in Figure 1, two 1-((1*H*-benzimidazol-1-yl)methyl)-1*H*-imidazole ligands and two monodeprotonated 1,3,5-benzenetricarboxylic acid anions coordinate to the Zn^II^ ion which is located on a twofold rotation axis. The Zn—O bond length is slightly shorter than the Zn—N bond length. The environment around the Zn^II^ ion can be best described as distorted tetrahedral. O—H···O, O—H···N, and N—H···O hydrogen bonds between solvent water/water molecules, between solvent water molecules and carboxylate O atoms, between carboxyl groups and solvent water molecules, between carboxyl groups and benzimidazole N atoms, between benzimidazole units and solvent water molecules of adjacent molecules consolidate the crystal packing (Figure 2).

### Experimental

A mixture of Zn(NO_3_)_2_ (0.1 mmol), 1-((1*H*-benzimidazol-1-yl)methyl)-1*H*-imidazole (0.1 mmol), 1,3,5-benzenetricarboxylic acid (0.1 mmol) and water (10 ml) was placed in a 25 ml Teflon-lined stainless steel vessel and heated at 120 °C for 72 h, then cooled to room temperature. Colourless crystals were obtained from the filtrate and dried in air.

### Refinement

H atoms bound to C atoms were positioned geometrically and refined as riding atoms, with C-H = 0.93 (aromatic) Å and 0.97 (CH_2_) Å. H atoms bound to N and O atoms were found from difference maps and refined with distance restraints of N-H = 0.86 Å and O-H = 0.82 (OH) Å and O-H = 0.85 (H_2_O) Å. All H atoms were refined with U_iso_(H) = 1.2 U_eq_(C,N,O).

### Crystal data

**Table:**

[Zn(C_9_H_5_O_6_)_2_(C_11_H_10_N_4_)_2_]·8H_2_O	*F*(000) = 2128
*M**_r_* = 1024.22	*D*_x_ = 1.489 Mg m^−^^3^
Monoclinic, *C*2/*c*	Mo *K*α radiation, λ = 0.71073 Å
Hall symbol: -C 2yc	Cell parameters from 6831 reflections
*a* = 20.870 (4) Å	θ = 1.7–27.9°
*b* = 15.008 (3) Å	µ = 0.63 mm^−^^1^
*c* = 15.472 (3) Å	*T* = 293 K
β = 109.51 (3)°	Prism, colourless
*V* = 4567.9 (16) Å^3^	0.18 × 0.15 × 0.14 mm
*Z* = 4	

### Data collection

**Table:**

Rigaku Saturn diffractometer	4127 independent reflections
Radiation source: fine-focus sealed tube	3898 reflections with *I* > 2σ(*I*)
graphite	*R*_int_ = 0.031
Detector resolution: 28.5714 pixels mm^-1^	θ_max_ = 25.3°, θ_min_ = 2.0°
ω scans	*h* = −25→25
Absorption correction: multi-scan (*CrystalClear*; Rigaku/MSC, 2004)	*k* = −17→18
*T*_min_ = 0.896, *T*_max_ = 0.917	*l* = −18→18
15325 measured reflections	

### Refinement

**Table:**

Refinement on *F*^2^	Primary atom site location: structure-invariant direct methods
Least-squares matrix: full	Secondary atom site location: difference Fourier map
*R*[*F*^2^ > 2σ(*F*^2^)] = 0.054	Hydrogen site location: inferred from neighbouring sites
*wR*(*F*^2^) = 0.158	H-atom parameters constrained
*S* = 1.07	*w* = 1/[σ^2^(*F*_o_^2^) + (0.0953*P*)^2^ + 7.0031*P*] where *P* = (*F*_o_^2^ + 2*F*_c_^2^)/3
4127 reflections	(Δ/σ)_max_ < 0.001
314 parameters	Δρ_max_ = 1.08 e Å^−^^3^
0 restraints	Δρ_min_ = −0.45 e Å^−^^3^

### Special details

**Table:**

Geometry. All e.s.d.'s (except the e.s.d. in the dihedral angle between two l.s. planes) are estimated using the full covariance matrix. The cell e.s.d.'s are taken into account individually in the estimation of e.s.d.'s in distances, angles and torsion angles; correlations between e.s.d.'s in cell parameters are only used when they are defined by crystal symmetry. An approximate (isotropic) treatment of cell e.s.d.'s is used for estimating e.s.d.'s involving l.s. planes.
Refinement. Refinement of *F*^2^ against ALL reflections. The weighted *R*-factor *wR* and goodness of fit *S* are based on *F*^2^, conventional *R*-factors *R* are based on *F*, with *F* set to zero for negative *F*^2^. The threshold expression of *F*^2^ > σ(*F*^2^) is used only for calculating *R*-factors(gt) *etc*. and is not relevant to the choice of reflections for refinement. *R*-factors based on *F*^2^ are statistically about twice as large as those based on *F*, and *R*- factors based on ALL data will be even larger.

### Fractional atomic coordinates and isotropic or equivalent isotropic displacement parameters (Å^2^)

**Table:**

	*x*	*y*	*z*	*U*_iso_*/*U*_eq_	
Zn1	1.0000	0.47361 (3)	0.7500	0.02673 (18)	
N1	1.00685 (12)	0.54973 (17)	0.85945 (17)	0.0303 (5)	
N2	1.04718 (12)	0.60129 (16)	0.99936 (16)	0.0295 (5)	
N3	1.20973 (13)	0.63838 (16)	1.09909 (17)	0.0323 (6)	
N4	1.15439 (13)	0.75634 (17)	1.11874 (19)	0.0368 (6)	
H4	1.1219	0.7891	1.1235	0.044*	
O1	0.91112 (10)	0.41744 (14)	0.70946 (15)	0.0353 (5)	
O2	0.95138 (12)	0.31291 (17)	0.64061 (18)	0.0486 (6)	
O3	0.67237 (16)	0.42512 (17)	0.6806 (3)	0.0685 (9)	
H3	0.6539	0.4603	0.6391	0.082*	
O4	0.60726 (13)	0.3058 (2)	0.6317 (3)	0.0730 (10)	
O5	0.70841 (13)	0.02986 (15)	0.5653 (3)	0.0604 (8)	
H5	0.7129	−0.0244	0.5703	0.073*	
O6	0.81799 (15)	0.02479 (16)	0.5819 (3)	0.0655 (9)	
O7	0.03572 (17)	0.8297 (2)	0.1368 (3)	0.0841 (11)	
H7A	0.0096	0.7939	0.1517	0.101*	
H7B	0.0500	0.8761	0.1688	0.101*	
O8	0.5834 (2)	0.5183 (3)	0.7433 (3)	0.1037 (14)	
H8A	0.5724	0.4636	0.7401	0.124*	
H8B	0.6017	0.5138	0.8011	0.124*	
O9	0.4962 (3)	0.3677 (5)	0.6555 (6)	0.214 (4)	
H9A	0.5130	0.3746	0.6129	0.256*	
H9B	0.4592	0.3966	0.6428	0.256*	
O10	0.4214 (6)	0.4833 (11)	0.5182 (7)	0.417 (11)	
H10A	0.4442	0.4475	0.5597	0.501*	
H10B	0.3822	0.4733	0.5214	0.501*	
C1	1.05420 (15)	0.53995 (19)	0.9405 (2)	0.0303 (6)	
H1A	1.0878	0.4964	0.9546	0.036*	
C2	0.96815 (16)	0.6220 (2)	0.8680 (2)	0.0381 (7)	
H2A	0.9311	0.6451	0.8215	0.046*	
C3	0.99263 (16)	0.6536 (2)	0.9542 (2)	0.0396 (8)	
H3A	0.9758	0.7015	0.9782	0.048*	
C4	1.09106 (16)	0.6115 (2)	1.0948 (2)	0.0344 (7)	
H4A	1.1063	0.5531	1.1204	0.041*	
H4B	1.0650	0.6377	1.1298	0.041*	
C5	1.15158 (15)	0.6686 (2)	1.10402 (19)	0.0302 (6)	
C6	1.21887 (16)	0.7856 (2)	1.1250 (2)	0.0347 (7)	
C7	1.24868 (19)	0.8692 (2)	1.1402 (3)	0.0483 (9)	
H7C	1.2251	0.9194	1.1480	0.058*	
C8	1.3160 (2)	0.8734 (3)	1.1431 (3)	0.0519 (9)	
H8C	1.3381	0.9282	1.1529	0.062*	
C9	1.35128 (18)	0.7980 (3)	1.1318 (3)	0.0506 (9)	
H9C	1.3964	0.8037	1.1353	0.061*	
C10	1.32114 (17)	0.7154 (2)	1.1157 (2)	0.0419 (8)	
H10C	1.3446	0.6653	1.1074	0.050*	
C11	1.25365 (15)	0.7105 (2)	1.1124 (2)	0.0327 (7)	
C12	0.90560 (15)	0.3445 (2)	0.6650 (2)	0.0314 (6)	
C13	0.83856 (15)	0.2976 (2)	0.64489 (19)	0.0297 (6)	
C14	0.78386 (15)	0.3418 (2)	0.6558 (2)	0.0332 (7)	
H14A	0.7884	0.4009	0.6749	0.040*	
C15	0.72163 (16)	0.2984 (2)	0.6385 (2)	0.0357 (7)	
C16	0.71589 (16)	0.2091 (2)	0.6120 (2)	0.0363 (7)	
H16A	0.6747	0.1797	0.6007	0.044*	
C17	0.77091 (16)	0.1639 (2)	0.6024 (2)	0.0346 (7)	
C18	0.83224 (16)	0.2091 (2)	0.6178 (2)	0.0344 (7)	
H18A	0.8691	0.1796	0.6098	0.041*	
C19	0.66265 (17)	0.3463 (2)	0.6498 (3)	0.0439 (8)	
C20	0.76632 (18)	0.0659 (2)	0.5806 (3)	0.0435 (8)	

### Atomic displacement parameters (Å^2^)

**Table:**

	*U*^11^	*U*^22^	*U*^33^	*U*^12^	*U*^13^	*U*^23^
Zn1	0.0215 (3)	0.0258 (3)	0.0320 (3)	0.000	0.0078 (2)	0.000
N1	0.0237 (12)	0.0282 (12)	0.0380 (14)	−0.0005 (10)	0.0089 (10)	−0.0032 (11)
N2	0.0276 (12)	0.0287 (12)	0.0324 (13)	−0.0035 (10)	0.0104 (10)	−0.0038 (10)
N3	0.0291 (13)	0.0262 (12)	0.0383 (14)	−0.0012 (10)	0.0070 (11)	0.0028 (11)
N4	0.0317 (14)	0.0321 (14)	0.0479 (16)	−0.0030 (11)	0.0152 (12)	−0.0087 (12)
O1	0.0294 (11)	0.0326 (11)	0.0417 (12)	−0.0077 (9)	0.0090 (9)	−0.0042 (9)
O2	0.0372 (13)	0.0513 (15)	0.0651 (16)	−0.0110 (11)	0.0276 (12)	−0.0135 (12)
O3	0.0585 (18)	0.0375 (15)	0.119 (3)	−0.0020 (13)	0.0428 (18)	−0.0142 (16)
O4	0.0347 (14)	0.0558 (17)	0.129 (3)	−0.0097 (13)	0.0275 (16)	−0.0182 (18)
O5	0.0418 (15)	0.0241 (12)	0.111 (3)	−0.0081 (10)	0.0193 (15)	−0.0041 (13)
O6	0.0515 (17)	0.0367 (15)	0.115 (3)	−0.0043 (12)	0.0364 (17)	−0.0187 (14)
O7	0.078 (2)	0.065 (2)	0.129 (3)	−0.0150 (17)	0.060 (2)	−0.018 (2)
O8	0.100 (3)	0.089 (3)	0.111 (3)	0.001 (2)	0.020 (3)	0.001 (2)
O9	0.117 (5)	0.204 (7)	0.321 (10)	0.020 (5)	0.074 (6)	−0.060 (7)
O10	0.317 (14)	0.76 (3)	0.254 (12)	0.332 (17)	0.204 (12)	0.243 (16)
C1	0.0241 (14)	0.0280 (15)	0.0376 (16)	−0.0019 (11)	0.0087 (12)	−0.0011 (12)
C2	0.0307 (16)	0.0373 (17)	0.0415 (18)	0.0074 (13)	0.0055 (13)	−0.0038 (14)
C3	0.0378 (17)	0.0364 (17)	0.0443 (18)	0.0087 (14)	0.0135 (15)	−0.0062 (14)
C4	0.0345 (16)	0.0372 (16)	0.0318 (15)	−0.0090 (13)	0.0113 (13)	−0.0024 (13)
C5	0.0302 (15)	0.0292 (15)	0.0298 (15)	−0.0035 (12)	0.0083 (12)	−0.0002 (12)
C6	0.0341 (16)	0.0323 (16)	0.0349 (16)	−0.0050 (13)	0.0077 (13)	−0.0040 (13)
C7	0.053 (2)	0.0324 (18)	0.060 (2)	−0.0087 (16)	0.0192 (18)	−0.0079 (16)
C8	0.051 (2)	0.043 (2)	0.058 (2)	−0.0209 (18)	0.0141 (18)	−0.0030 (17)
C9	0.0346 (18)	0.061 (2)	0.053 (2)	−0.0129 (17)	0.0102 (16)	0.0041 (18)
C10	0.0336 (17)	0.0413 (19)	0.049 (2)	0.0004 (14)	0.0110 (15)	0.0042 (15)
C11	0.0312 (15)	0.0283 (15)	0.0347 (16)	−0.0033 (12)	0.0059 (12)	0.0020 (12)
C12	0.0313 (15)	0.0315 (15)	0.0306 (15)	−0.0058 (12)	0.0092 (12)	0.0026 (12)
C13	0.0292 (15)	0.0295 (15)	0.0282 (14)	−0.0068 (12)	0.0065 (12)	0.0010 (12)
C14	0.0341 (16)	0.0243 (14)	0.0388 (17)	−0.0066 (12)	0.0092 (13)	−0.0024 (12)
C15	0.0303 (16)	0.0310 (16)	0.0436 (18)	−0.0041 (13)	0.0093 (13)	−0.0014 (13)
C16	0.0304 (16)	0.0294 (16)	0.0460 (18)	−0.0070 (13)	0.0084 (13)	0.0007 (13)
C17	0.0315 (16)	0.0273 (15)	0.0429 (18)	−0.0067 (12)	0.0097 (13)	−0.0013 (13)
C18	0.0309 (16)	0.0330 (16)	0.0405 (17)	−0.0047 (13)	0.0137 (13)	−0.0020 (13)
C19	0.0345 (18)	0.0325 (17)	0.063 (2)	−0.0033 (14)	0.0139 (16)	−0.0040 (16)
C20	0.0409 (19)	0.0302 (17)	0.058 (2)	−0.0065 (15)	0.0153 (16)	−0.0036 (15)

### Geometric parameters (Å, °)

**Table:**

Zn1—O1	1.941 (2)	C1—H1A	0.9300
Zn1—O1^i^	1.941 (2)	C2—C3	1.344 (5)
Zn1—N1	2.008 (2)	C2—H2A	0.9300
Zn1—N1^i^	2.008 (2)	C3—H3A	0.9300
N1—C1	1.320 (4)	C4—C5	1.493 (4)
N1—C2	1.385 (4)	C4—H4A	0.9700
N2—C1	1.338 (4)	C4—H4B	0.9700
N2—C3	1.367 (4)	C6—C7	1.384 (5)
N2—C4	1.463 (4)	C6—C11	1.390 (4)
N3—C5	1.322 (4)	C7—C8	1.392 (5)
N3—C11	1.388 (4)	C7—H7C	0.9300
N4—C5	1.334 (4)	C8—C9	1.392 (6)
N4—C6	1.388 (4)	C8—H8C	0.9300
N4—H4	0.8600	C9—C10	1.375 (5)
O1—C12	1.278 (4)	C9—H9C	0.9300
O2—C12	1.233 (4)	C10—C11	1.394 (5)
O3—C19	1.266 (4)	C10—H10C	0.9300
O3—H3	0.8200	C12—C13	1.503 (4)
O4—C19	1.253 (4)	C13—C14	1.378 (4)
O5—C20	1.272 (4)	C13—C18	1.386 (4)
O5—H5	0.8200	C14—C15	1.396 (4)
O6—C20	1.236 (4)	C14—H14A	0.9300
O7—H7A	0.8500	C15—C16	1.395 (4)
O7—H7B	0.8492	C15—C19	1.485 (5)
O8—H8A	0.8501	C16—C17	1.384 (5)
O8—H8B	0.8502	C16—H16A	0.9300
O9—H9A	0.8500	C17—C18	1.397 (4)
O9—H9B	0.8501	C17—C20	1.505 (4)
O10—H10A	0.8502	C18—H18A	0.9300
O10—H10B	0.8498		
			
O1—Zn1—O1^i^	128.52 (13)	N4—C6—C11	105.8 (3)
O1—Zn1—N1	108.11 (10)	C6—C7—C8	116.0 (3)
O1^i^—Zn1—N1	100.57 (10)	C6—C7—H7C	122.0
O1—Zn1—N1^i^	100.57 (10)	C8—C7—H7C	122.0
O1^i^—Zn1—N1^i^	108.11 (10)	C7—C8—C9	121.9 (3)
N1—Zn1—N1^i^	110.65 (15)	C7—C8—H8C	119.0
C1—N1—C2	105.8 (3)	C9—C8—H8C	119.0
C1—N1—Zn1	123.9 (2)	C10—C9—C8	121.8 (3)
C2—N1—Zn1	130.3 (2)	C10—C9—H9C	119.1
C1—N2—C3	108.0 (2)	C8—C9—H9C	119.1
C1—N2—C4	125.9 (3)	C9—C10—C11	116.7 (3)
C3—N2—C4	126.2 (3)	C9—C10—H10C	121.6
C5—N3—C11	107.3 (3)	C11—C10—H10C	121.6
C5—N4—C6	108.1 (3)	N3—C11—C6	107.7 (3)
C5—N4—H4	125.9	N3—C11—C10	131.0 (3)
C6—N4—H4	125.9	C6—C11—C10	121.3 (3)
C12—O1—Zn1	116.8 (2)	O2—C12—O1	123.7 (3)
C19—O3—H3	109.5	O2—C12—C13	121.3 (3)
C20—O5—H5	109.5	O1—C12—C13	115.0 (3)
H7A—O7—H7B	119.4	C14—C13—C18	119.9 (3)
H8A—O8—H8B	90.1	C14—C13—C12	120.1 (3)
H9A—O9—H9B	109.5	C18—C13—C12	119.9 (3)
H10A—O10—H10B	98.7	C13—C14—C15	120.5 (3)
N1—C1—N2	110.7 (3)	C13—C14—H14A	119.7
N1—C1—H1A	124.7	C15—C14—H14A	119.7
N2—C1—H1A	124.7	C16—C15—C14	119.2 (3)
C3—C2—N1	109.3 (3)	C16—C15—C19	120.4 (3)
C3—C2—H2A	125.4	C14—C15—C19	120.4 (3)
N1—C2—H2A	125.4	C17—C16—C15	120.6 (3)
C2—C3—N2	106.3 (3)	C17—C16—H16A	119.7
C2—C3—H3A	126.9	C15—C16—H16A	119.7
N2—C3—H3A	126.9	C16—C17—C18	119.3 (3)
N2—C4—C5	112.4 (2)	C16—C17—C20	120.7 (3)
N2—C4—H4A	109.1	C18—C17—C20	119.9 (3)
C5—C4—H4A	109.1	C13—C18—C17	120.4 (3)
N2—C4—H4B	109.1	C13—C18—H18A	119.8
C5—C4—H4B	109.1	C17—C18—H18A	119.8
H4A—C4—H4B	107.9	O4—C19—O3	123.9 (3)
N3—C5—N4	111.1 (3)	O4—C19—C15	118.6 (3)
N3—C5—C4	124.1 (3)	O3—C19—C15	117.5 (3)
N4—C5—C4	124.8 (3)	O6—C20—O5	124.1 (3)
C7—C6—N4	132.0 (3)	O6—C20—C17	119.6 (3)
C7—C6—C11	122.2 (3)	O5—C20—C17	116.3 (3)
			
O1—Zn1—N1—C1	121.9 (2)	C5—N3—C11—C10	178.6 (3)
O1^i^—Zn1—N1—C1	−14.8 (3)	C7—C6—C11—N3	179.9 (3)
N1^i^—Zn1—N1—C1	−128.9 (3)	N4—C6—C11—N3	0.0 (3)
O1—Zn1—N1—C2	−60.7 (3)	C7—C6—C11—C10	0.8 (5)
O1^i^—Zn1—N1—C2	162.6 (3)	N4—C6—C11—C10	−179.1 (3)
N1^i^—Zn1—N1—C2	48.5 (3)	C9—C10—C11—N3	−178.9 (3)
O1^i^—Zn1—O1—C12	−34.72 (19)	C9—C10—C11—C6	−0.1 (5)
N1—Zn1—O1—C12	−155.2 (2)	Zn1—O1—C12—O2	−6.1 (4)
N1^i^—Zn1—O1—C12	88.8 (2)	Zn1—O1—C12—C13	172.90 (18)
C2—N1—C1—N2	0.5 (3)	O2—C12—C13—C14	−166.9 (3)
Zn1—N1—C1—N2	178.40 (19)	O1—C12—C13—C14	14.1 (4)
C3—N2—C1—N1	−0.2 (3)	O2—C12—C13—C18	14.9 (4)
C4—N2—C1—N1	−179.1 (3)	O1—C12—C13—C18	−164.2 (3)
C1—N1—C2—C3	−0.6 (4)	C18—C13—C14—C15	−0.9 (5)
Zn1—N1—C2—C3	−178.3 (2)	C12—C13—C14—C15	−179.2 (3)
N1—C2—C3—N2	0.5 (4)	C13—C14—C15—C16	1.5 (5)
C1—N2—C3—C2	−0.2 (4)	C13—C14—C15—C19	−179.6 (3)
C4—N2—C3—C2	178.7 (3)	C14—C15—C16—C17	−0.4 (5)
C1—N2—C4—C5	86.6 (4)	C19—C15—C16—C17	−179.3 (3)
C3—N2—C4—C5	−92.1 (4)	C15—C16—C17—C18	−1.2 (5)
C11—N3—C5—N4	0.7 (3)	C15—C16—C17—C20	175.5 (3)
C11—N3—C5—C4	−178.9 (3)	C14—C13—C18—C17	−0.7 (5)
C6—N4—C5—N3	−0.8 (4)	C12—C13—C18—C17	177.5 (3)
C6—N4—C5—C4	178.9 (3)	C16—C17—C18—C13	1.8 (5)
N2—C4—C5—N3	−87.7 (4)	C20—C17—C18—C13	−175.0 (3)
N2—C4—C5—N4	92.7 (4)	C16—C15—C19—O4	−2.6 (5)
C5—N4—C6—C7	−179.5 (4)	C14—C15—C19—O4	178.5 (3)
C5—N4—C6—C11	0.5 (3)	C16—C15—C19—O3	174.8 (4)
N4—C6—C7—C8	179.3 (3)	C14—C15—C19—O3	−4.1 (5)
C11—C6—C7—C8	−0.6 (5)	C16—C17—C20—O6	−172.3 (4)
C6—C7—C8—C9	−0.3 (6)	C18—C17—C20—O6	4.4 (5)
C7—C8—C9—C10	1.0 (6)	C16—C17—C20—O5	4.9 (5)
C8—C9—C10—C11	−0.8 (5)	C18—C17—C20—O5	−178.4 (3)
C5—N3—C11—C6	−0.4 (3)		

Symmetry codes: (i) −*x*+2, *y*, −*z*+3/2.

### Hydrogen-bond geometry (Å, °)

**Table:**

*D*—H···*A*	*D*—H	H···*A*	*D*···*A*	*D*—H···*A*
O8—H8A···O9	0.85	2.22	2.936 (8)	142.
O9—H9A···O4	0.85	2.15	2.634 (7)	115.
O9—H9B···O10	0.85	2.24	2.783 (12)	122.
O10—H10A···O9	0.85	1.93	2.783 (12)	179.
N4—H4···O7^ii^	0.86	1.97	2.808 (4)	163.
O3—H3···O10^iii^	0.82	2.56	3.341 (10)	159.
O5—H5···N3^iv^	0.82	1.77	2.577 (3)	165.
O7—H7A···O2^v^	0.85	1.98	2.784 (4)	156.
O7—H7B···O8^vi^	0.85	1.95	2.796 (5)	179.
O8—H8B···O6^vii^	0.85	2.02	2.799 (6)	152.
O10—H10B···O6^viii^	0.85	2.03	2.728 (9)	139.

Symmetry codes: (ii) *x*+1, *y*, *z*+1; (iii) −*x*+1, −*y*+1, −*z*+1; (iv) *x*−1/2, −*y*+1/2, *z*−1/2; (v) *x*−1, −*y*+1, *z*−1/2; (vi) *x*−1/2, −*y*+3/2, *z*−1/2; (vii) −*x*+3/2, *y*+1/2, −*z*+3/2; (viii) *x*−1/2, *y*+1/2, *z*.
